# Vaccine‐induced binding and neutralizing antibodies against Omicron 6 months after a homologous BNT162b2 booster

**DOI:** 10.1002/jmv.28164

**Published:** 2022-10-01

**Authors:** Julien Favresse, Constant Gillot, Jean‐Louis Bayart, Clara David, Germain Simon, Loris Wauthier, Mélanie Closset, Jean‐Michel Dogné, Jonathan Douxfils

**Affiliations:** ^1^ Department of Laboratory Medicine Clinique St‐Luc Bouge Namur Belgium; ^2^ Department of Pharmacy, Namur Research Institute for LIfe Sciences University of Namur Namur Belgium; ^3^ Department of Laboratory Medicine Clinique St‐Pierre Ottignies‐Louvain‐la‐Neuve Belgium; ^4^ Qualiblood s.a. Rue du Séminaire 20A Namur Belgium; ^5^ Department of Laboratory Medicine Université catholique de Louvain, CHU UCL Namur, Yvoir Namur Belgium

**Keywords:** binding antibodies, BNT162b2, COVID‐19, mRNA vaccine, neutralizing antibodies, Omicron, SARS‐CoV‐2, vaccine efficacy

## Abstract

Evidence about the long‐term persistence of the booster‐mediated immunity against Omicron is mandatory for pandemic management and deployment of vaccination strategies. A total of 155 healthcare professionals (104 COVID‐19 naive and 51 with a history of SARS‐CoV‐2 infection) received a homologous BNT162b2 booster. Binding antibodies against the spike protein and neutralizing antibodies against Omicron were measured at several time points before and up to 6 months after the booster. Geometric mean titers of measured antibodies were correlated to vaccine efficacy (VE) against symptomatic disease. Compared to the highest response, a significant 10.2‐ and 11.5‐fold decrease in neutralizing titers was observed after 6 months in participants with and without history of SARS‐CoV‐2 infection. A corresponding 2.5‐ and 2.9‐fold decrease in binding antibodies was observed. The estimated T_1/2_ of neutralizing antibodies in participants with and without history of SARS‐CoV‐2 infection was 42 (95% confidence interval [CI]: 25–137) and 36 days (95% CI: 25–65). Estimated T_1/2_ were longer for binding antibodies: 168 (95% CI: 116–303) and 139 days (95% CI: 113–180), respectively. Both binding and neutralizing antibodies were strongly correlated to VE (*r* = 0.83 and 0.89). However, binding and neutralizing antibodies were modestly correlated, and a high proportion of subjects (36.7%) with high binding antibody titers (i.e., >8434 BAU/ml) did not have neutralizing activity. A considerable decay of the humoral response was observed 6 months after the booster, and was strongly correlated with VE. Our study also shows that commercial assays available in clinical laboratories might require adaptation to better predict neutralization in the Omicron era.

## 1 INTRODUCTION

Early efficacy trials and real‐world data on the BNT162b2 messenger RNA (mRNA) vaccine confirmed its high effectiveness in reducing laboratory‐confirmed infection, COVID‐19 hospitalization and death.^1^, ^2^, ^3^, ^4^ Nevertheless, a gradual decline in vaccine efficacy (VE) over time has been observed within the first months after the initial two‐dose regimen.^4^, ^5^, ^6^, ^7^ This waned efficacy was consistent with the decrease of neutralizing antibodies observed by multiple independent studies^8^, ^9^, ^10^ supporting neutralizing antibodies as a strong correlate of COVID‐19 protection.^11^, ^12^, ^13^, ^14^

Moreover, since the beginning of the pandemic, several mutations occurred in the SARS‐CoV‐2 genome leading the different lineages of the virus.^15^ Five of these lineages have been designated as a variant of concern (VOC) by the World Health Organization, namely the Alpha, Beta, Gamma, Delta and Omicron variants.^16^ Discovered in November 2021, the Omicron lineage is to date the leading variant over the world.^15^ This variant is characterized by 32 dominant mutations in the spike (S) protein, 15 of which are located in the receptor‐binding domain (RBD) conferring an increased transmissibility and a considerable immune escape from acquired protection through SARS‐CoV‐2 vaccination or a previous infection.^17^, ^18^, ^19^, ^20^, ^21^, ^22^, ^23^, ^24^ Currently, Omicron is largely dominant and several subvariants have emerged including BA.2, BA.2.12.1, BA.4, and BA.5.^15^ All these subvariants have also demonstrated a considerable escape to acquired immunity.^20^, ^25^, ^26^

The current BNT162b2 vaccine, which has been elaborated on the sequence of the wild‐type SARS‐CoV‐2,^27^ has been shown to be less effective against Omicron compared to other VOCs^28^, ^29^, ^30^, ^31^ and the VE also waned over time to reach zero to 22.3% 6 months after the second BNT162b2 dose.^5^, ^7^, ^30^, ^32^, ^33^ With the decreased efficacy of vaccines over time and the emergence of highly transmissible SARS‐CoV‐2 variants that escape neutralization, many countries have deployed third doses of COVID‐19 vaccines.

The administration of a homologous BNT162b2 booster dose increased the VE to 58.9% (interquartile range  [IQR] = 52.7%–63.3%) within 2–4 weeks.^7^, ^30^, ^32^, ^33^, ^34^ This increase was consistent with the rise of binding (median fold increase = 26.8; IQR = 13.6–51.7)^35^, ^36^, ^37^ and neutralizing antibodies (median fold increase = 27.3; IQR = 10.2–52.7).^18^, ^19^, ^38^, ^39^, ^40^, ^41^ However, a waning of protection against symptomatic diseases was rapidly observed 8–14 weeks after the booster (median VE = 37.9%; IQR = 24.6%–45.1%).^7^, ^30^, ^33^ Although protection against severe COVID‐19 remains higher, the Center for Disease Control and Prevention reported that after receiving both 2 and 3 doses, the VE was lower during the Omicron‐predominant than during the Delta‐predominant period at all time points evaluated.^42^

Evidence about the long‐term persistence of the booster‐mediated immunity against Omicron is crucial knowledge for pandemic response. The aim of this study was to evaluate 6‐month humoral response in a cohort of healthcare workers (HCW) who received the homologous BNT162b2 booster.

## 2 MATERIALS AND METHODS

### 2.1 Study design and participants

The CRO‐VAX HCP study is a Belgian multicenter, prospective, and interventional study that was designed to assess the antibody response in a population of HCW from 18 to 65 years of age having received two doses of the BNT162b2 mRNA COVID‐19 vaccine (Comirnaty®, Pfizer‐BioNTech).^9^, ^43^, ^44^ The study was approved by a central ethical committee (approval number: 2020‐006149‐21) and a total of 231 participants were initially enrolled. Participants received the first vaccine dose between 18 January and 17 February 2021. The second dose was then administered 21 days after the first one. Thereafter, participants were proposed to receive a homologous booster that was administered between 8 November 2021 and 31 January 2022. A total of 155 volunteers (67.1%) agreed to receive the booster and to pursue the study. Blood was collected at 7 different time points for the evaluation of the booster‐induced immunity, i.e., maximum 2 days before the booster injection and after 7, 14, 28, 56, 90, and 180 days (i.e., 6 months). Blood samplings performed earlier or later than the expected blood collection times were allowed with a maximal allowed percentage of 10% (i.e., 180 days = 18 days). Volunteers that missed a blood sampling were not excluded from the analysis. Subjects having positive antibodies against the SARS‐CoV‐2 nucleocapsid (NCP) antigen before the booster were considered seropositive (i.e., history of SARS‐CoV‐2 infection) while the others were COVID‐19 naive and classified as seronegative. Anti‐NCP were also used to document the development of a breakthrough infection during the study follow‐up.

### 2.2 Analytical procedures

#### 2.2.1 Neutralizing antibodies

A pseudovirus‐neutralization test was used to assess the neutralization potency of BNT162b2‐elicited antibodies against the Omicron BA.1 variant. Pseudoviruses were from E‐enzyme. SARS‐CoV‐2 Pseudoviral Particles are replication‐deficient Maloneymurine leukemia virus (MLV or MuLV) pseudotyped with the SARS‐CoV‐2 spike protein carrying the Omicron B1.1.529 genotype. They also contain the open reading frame for firefly luciferase as a reporter. Briefly, HEK293T hACE2 cells were seeded at the density of 8500 cells/well in a white 384‐well cell culture plate. The sera used are heat‐inactivated by a water bath at 54°C for 30 min and then serially diluted in a culture medium (Dulbecco's modified Eagle medium) supplemented with 10% of fetal bovine serum. Thereafter, samples are mixed in a 1:4 ratio with pseudovirus and incubated for 2 h at 37°C. This mixture is added to the cells and incubated for 48 h at 37°C. The reading is done by adding a reagent to measure the activity of luciferase which is proportional to the cells infected by the pseudovirus. Raw data obtained in relative luminescence units are normalized to the positive control where cells are incubated with pseudovirus in the absence of serum. The antibody titer is determined as the dilution of serum at which 50% of the infectivity is inhibited (IC_50_) as determined by a nonlinear sigmoid regression model. A sample with a titer of less than 1/20 is considered negative.^45^, ^46^

#### 2.2.2 Binding antibodies

Binding antibodies against the RBD of the S1 subunit of the SARS‐CoV‐2 spike protein were measured by the Elecsys Anti‐SARS‐CoV‐2 S assay that measured total antibodies (Roche Diagnostics) with a positivity cut‐off of 0.8 BAU/ml. An automatic dilution of 1/100 at >250 BAU/ml was performed by the analyzer to extend the measurement domain up to 25 000 BAU/ml. Additionally, total antibodies against the SARS‐CoV‐2 NCP (Roche Diagnostics) were measured using the Elecsys Anti‐SARS‐CoV‐2 assay. Results above 0.165 cut‐off index were considered positive.^47^

### 2.3 Statistical analyses

Median and IQR were used to present demographic data and geometric mean titers (GMT) and 95% confidence intervals (95% CI) for binding and neutralizing antibodies. The between‐group differences were tested using a Tukey multiple comparison test with a multiple testing correction.

The kinetic models for binding and neutralizing antibodies were calculated using the following equation and using nonlog transformed data:

$$\frac{\left(a\times b\right)}{\left[(a-b\times \mathrm{basal}\;\mathrm{response})\times {\mathrm{Exp}}^{\left(-\mathrm{Days}\;\mathrm{since}\;\mathrm{vaccination}\times c\right)}]+[b\times {\mathrm{Exp}}^{\left(\mathrm{Days}\;\mathrm{since}\;\mathrm{vaccination}\times d\right)}\right]}$$

Where “a” stands for the maximal antibody response, “b” stands for the baseline response, “c” for the antibody production rate and “d” for the antibody elimination rate.

The elimination rate was obtained from the model which permitted the calculation of the half‐life (T_1/2_). The time to maximal concentration (T_max_) and the mean time needed to cross the positivity threshold were also determined based on this model. In each patient, time points corresponding to the breakthrough infections were removed from kinetics to avoid rebound response bias.

Pearson's correlation was performed for the comparison between binding antibodies and neutralization titer. A Cohen's kappa agreement test was also calculated, and a receiver operating characteristic (ROC) curve analysis was performed to identify the best cut‐off to predict the neutralizing of the Omicron BA.1 variant (>1/20) using the binding antibody assay.

Spearman's rank correlation was performed for the comparison between log‐transformed geometric means of binding or neutralization antibodies and reported VE expressed in percentage against symptomatic disease. The VE was retrieved from the literature for the Omicron lineage only.^7^, ^29^, ^30^, ^31^, ^32^, ^34^, ^48^ Furthermore, only VE that concerned the homologous BNT162b2 booster administered to adults were included. The timings of blood collections were matched with those of published VE.

Statistical analyses were performed using GraphPad Prism 9.4.0 (GraphPad Software), JMP Pro 16.0.0 (JMP®, version 16.0.0. SAS Institute Inc.), and MedCalc Software (version 14.8.1). A *p* < 0.05 was considered significant.

## 3 RESULTS

### 3.1 Demographic data

A total of 155 HCW were included in the study. Among the participants, 112 (72.3%) were female (median age = 45 years; IQR =  36–54 years) and 39 (27.7%) were male (median age = 41 years; range = 29–57 years). Age was the same among gender (*p* = 0.27). A total of 104 subjects (67.1%) were COVID‐19 naive before booster administration while 51 (32.9%) had a previous history of infection. Age was not significantly different between groups (*p* = 0.36). The median time between first and third vaccine dose was 305 days (IQR = 294–310 days) and the median follow‐up time was 489 days (IQR = 475–498 days) since first dose. A total of 75 participants (48.4%) developed a breakthrough infection after the booster as evidenced by the new development or the rising of antibodies against the NCP.

### 3.2 Neutralizing antibodies against the Omicron BA.1 variant

**Figure 1:**
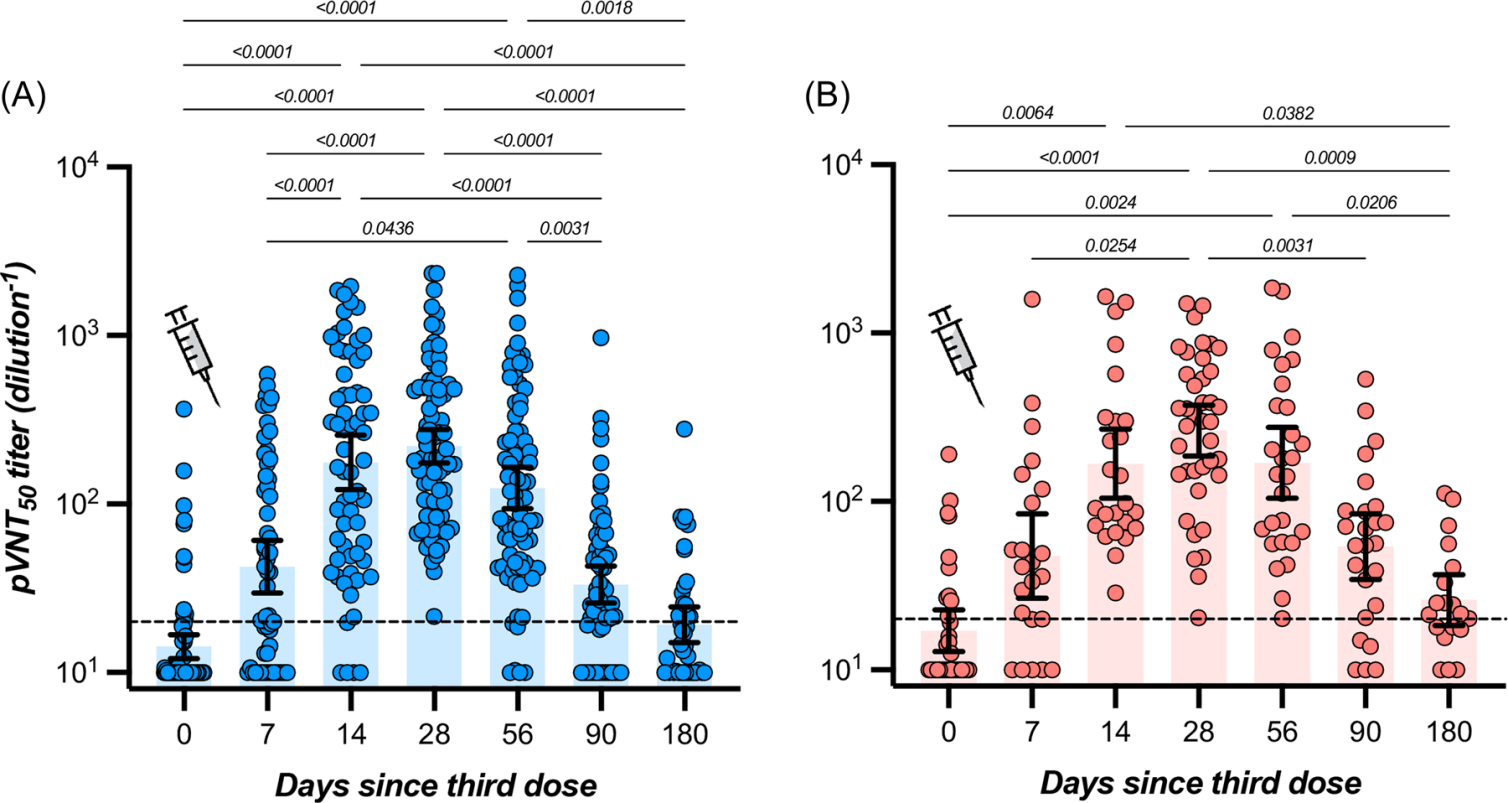
Fifty percent relative inhibition pseudovirus‐neutralization titers of sera from vaccine recipients, collected before and after the homologous BNT162b2 booster, with a 6‐month follow‐up. The SARS‐CoV‐2 pseudovirus bears the Omicron BA.1 variant S protein. The positivity cut‐off corresponds to a dilution titer of 1/20. The *blue color* corresponds to individuals that were never infected (A) and the *red color* to individuals that have a history of SARS‐CoV‐2 infection (B). Geometric means and 95% CI are represented. CI, confidence interval

In participants with no history of SARS‐CoV‐2 infection, the highest measured neutralizing capacity was reached at day 28 with a GMT of 221 (95% CI = 175–277), representing a 15.5‐fold increase from baseline (i.e., 14.3; 95% CI = 12.1–16.8). A continuous decrease was then observed up to day 180 with an observed GMT of 19.3 (95% CI = 15.1–24.6), which represents a 11.5‐fold decrease. At 6 months, the mean neutralizing titer was not significantly different from baseline (Table 1 and Figure 1). In participants with history of SARS‐CoV‐2 infection, the highest neutralizing capacity was also reached at day 28 with a GMT of 264 (95% CI = 186–373), corresponding to a 15.4‐fold increase from baseline (i.e., 17.1; 95% CI = 12.8–22.7). As observed in COVID‐19 naive individuals, a continuous decline was observed up to day 180 with a GMT of 26.0 (95% CI = 18.3–36.8), which represents a 10.2‐fold decrease. The mean titer at 6 months was comparable to baseline (Table 1 and Figure 1). For each time point, no significant differences were observed in individuals with or without history of SARS‐CoV‐2 infection (*p* > 0.05) (Table 1). The proportion of detectable Omicron‐specific neutralizing antibodies was low at baseline (16.0% and 29.4% for participants without or with history of SARS‐CoV‐2 infection, respectively) and progressively increased to achieve 100% at day 28 for COVID‐19 naive individuals and 100% between days 14 and 56 for past‐COVID‐19 subjects. Afterward, the seroprevalence progressively decreased to achieve 37.2% and 63.2% after 6 months in individuals without or with previous history of SARS‐CoV‐2 infection (Figure 1). The estimated T_1/2_ of neutralizing antibodies for COVID‐19 naive participants was 36 days (95% CI = 25–65 days). The T_max_ was estimated at 18 days (95% CI = 14–22 days). In previously infected subjects, the estimated T_1/2_ of neutralizing antibodies was 42 days (95% CI = 25–137 days) and the T_max_ was reached at 24 days (95% CI = 15–32 days). Estimations for T_1/2_ and T_max_ were not significantly different between groups. According to the model, a mean time of 182 days (95% CI = 118–234) in COVID‐19 naive participants and 214 days (95% CI = 110–297) in previously infected subjects would be needed to cross the dilution titer threshold of 1/20 (Figure 3A).

**Table 1:** Fifty percent relative inhibition pseudovirus‐neutralization titers and binding antibodies titers of sera from vaccine recipients, collected before and after the homologous BNT162b2 booster

	Never infected (*n* = 104)		History of infection (*n* = 51)		
	GMT (95% CI)	% pos. samples	GMT (95% CI)	% pos. samples	*p* value
pVNT_50_ titer (dilution^−1^)					
Before booster	14.3 (12.1–16.8)	16.0	17.1 (12.8–22.7)	29.4	>0.99
7 days	42.4 (29.6–60.8)	59.6	47.5 (26.7–84.3)	74.0	>0.99
14 days	177 (122–266)	92.3	168 (104–269)	100	0.91
28 days	221 (175–277)	100	264 (186–373)	100	>0.99
56 days	125 (94.0–165)	92.7	170 (105–275)	100	0.99
90 days	33.3 (25.8–42.9)	71.4	53.9 (34.4–84.4)	80.0	>0.99
180 days	19.3 (15.1–24.6)	37.2	26.0 (18.3–36.8)	63.2	>0.99
Binding antibodies (BAU/ml)					
Before booster	480 (407–566)	0.0	1999 (1590–2512)	6.1	<0.0001
7 days	14 879 (12 056–18 364)	86.6	15 842 (12 618–19 891)	91.9	>0.99
14 days	18 834 (17 295–20 509)	92.0	17 461 (15 028–20 288)	97.1	0.99
28 days	17 386 (15 834–19 090)	93.4	15 271 (13 241–17 613)	90.2	0.85
56 days	14 463 (13 002–16 088)	81.0	12 123 (9724–15 113)	68.8	>0.99
90 days	11 505 (9915–13 351)	73.4	9610 (7017–13 160)	62.5	>0.99
180 days	6508 (5080–8338)	38.6	6868 (4461–10 573)	52.6	>0.99

*Note*: The percentage of positive sera according to the assay considered are also represented. GMT stand for geometric mean titers. Positive cut‐offs were >20 dilution titer‐1 and >8434 BAU/ml for neutralizing and binding antibodies, respectively. The *p* value expresses the statistical difference between GMT of seronegative and seropositive persons.

Abbreviations: CI, confidence interval; pVNT, pseudovirus‐neutralization test.

### 3.3 Binding antibodies

**Figure 2:**
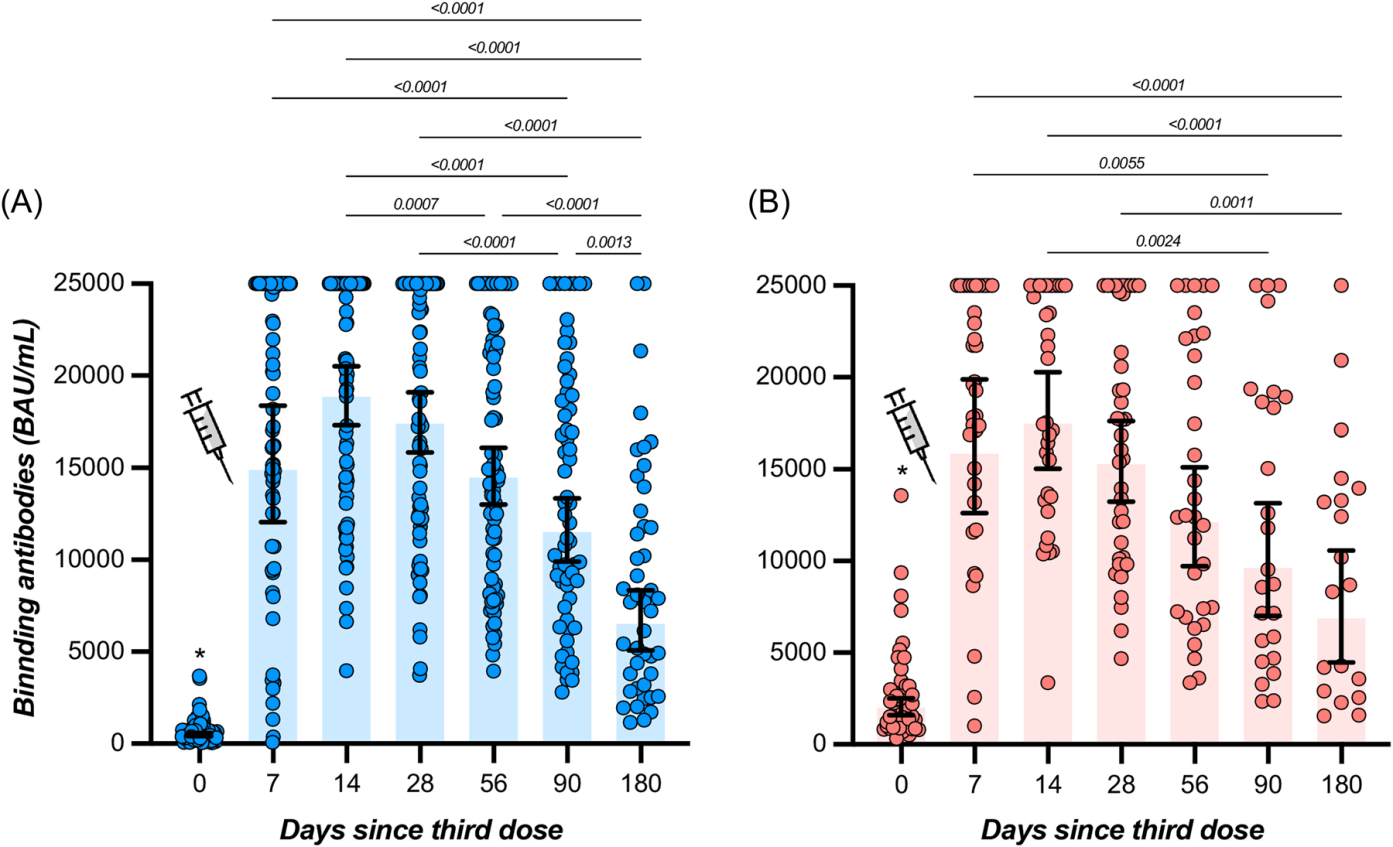
Binding antibodies of sera from vaccine recipients, collected before and after the homologous BNT162b2 booster, with a 6‐month follow‐up. The positivity cut‐off is 0.8 BAU/ml. The *blue color* corresponds to individuals that were never infected (A) and the *red color* to individuals that have a history of SARS‐CoV‐2 infection (B). Geometric means and 95% CI are represented. * = The time point at baseline (or “zero”) was significantly lower compared to other time points. CI, confidence interval

**Figure 3:**
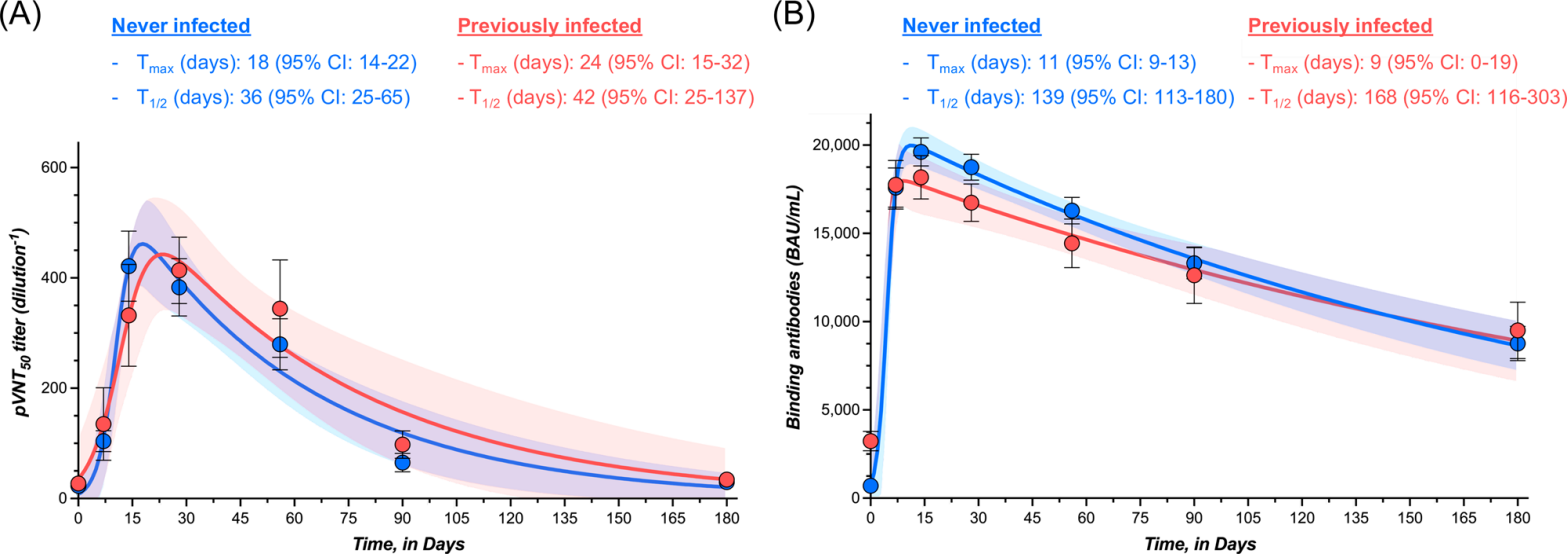
Kinetics models of (A) neutralizing antibodies against Omicron and (B) binding antibodies after the homologous BNT162b2 booster. Means plus/minus standard deviation are shown at the different time points. The *blue color* corresponds to individuals that were never infected and the *red color* to individuals that were previously infected with the SARS‐CoV‐2

**Figure 4:**
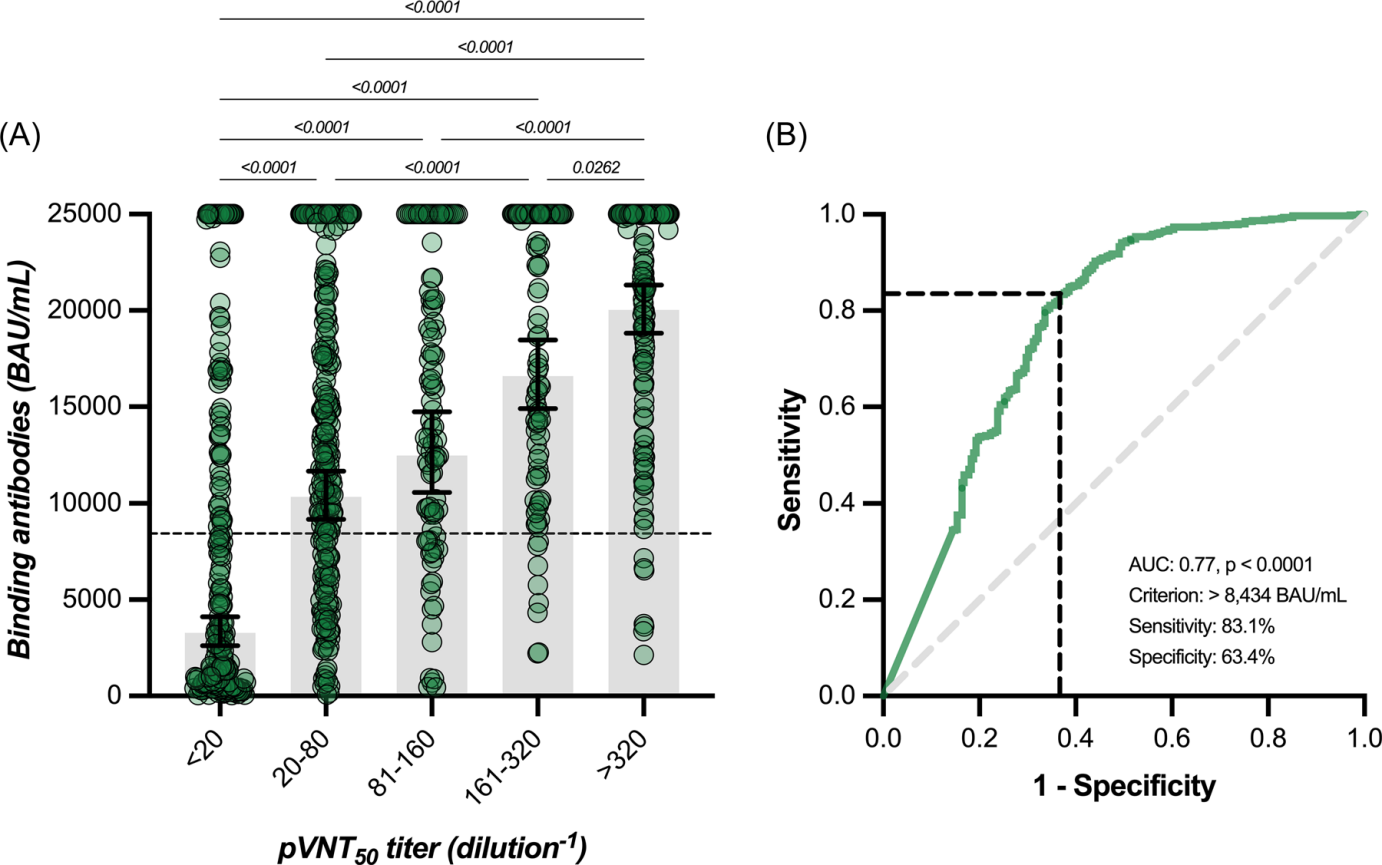
(A) Binding antibodies according to rank categories of neutralizing antibodies against the Omicron BA.1 variant. Geometric means and 95% CI are represented. (B) ROC curve analysis between binding antibodies (continuous variable) and neutralizing antibodies (i.e., >1/20 as the classification variable). The >8434 criterion (BAU/ml) corresponds to the best Youden index calculated. CI, confidence interval; ROC, receiver operating characteristic

In participants with no history of SARS‐CoV‐2 infection, the highest measured binding antibody response was reached at day 14 with a GMT of 18 834 BAU/ml (95% CI = 17 295–20 509), representing a 39.2‐fold rise from baseline (i.e., 480 BAU/ml; 95% CI = 407–566). A continuous decrease was then observed up to day 180 with an observed GMT of 6508 BAU/ml (95% CI = 5080–8338), which represents a 2.9‐fold decrease compared to day 14. Levels of binding antibodies at 6 months were higher compared to baseline (Table 1 and Figure 2). In participants that were previously infected, the highest binding antibody response was reached at day 14 with a GMT of 17 461 BAU/ml (95% CI = 15 028–20 288), corresponding to a 8.7‐fold increase from baseline (i.e., 1999 BAU/ml; 95% CI = 1590–2512). A continuous decline was observed up to day 180 with a GMT of 6868 BAU/ml (95% CI = 4461–10 573), which represents a 2.5‐fold decrease in binding antibody titers at 6 months. Six‐month titers were higher compared to baseline titers (Table 1 and Figure 2). Except at baseline (i.e., just before the administration of the booster), no significant differences were observed in individuals with or without history of SARS‐CoV‐2 infection (*p* > 0.05) (Table 1). All participants still had detectable positive binding antibodies 6 months after the booster (i.e., >0.8 BAU/ml). The estimated T_1/2_ of binding antibodies for COVID‐19 naive participants was 139 days (95% CI = 113–180 days) and the T_max_ was reached at 11 days (95% CI = 9–13 days). In previously infected subjects, the estimated T_1/2_ of binding antibodies was 168 days (95% CI = 116–303 days) and the T_max_ was reached at 9 days (95% CI = 0–19 days). Estimations for T_1/2_ and T_max_ were not significantly different between groups. According to the model, a mean time of 186 days (95% CI = 155–223) in COVID‐19 naive participants and 194 days (95% CI = 142–283) in previously infected subjects would be needed to cross the threshold of 8434 BAU/ml (Figure 3B). This threshold represents the binding antibody titer needed to ensure a neutralizing activity of 1/20 (Figure 4B).

### 3.4 Binding antibodies versus neutralizing antibodies and correlation to VE

**Figure 5:**
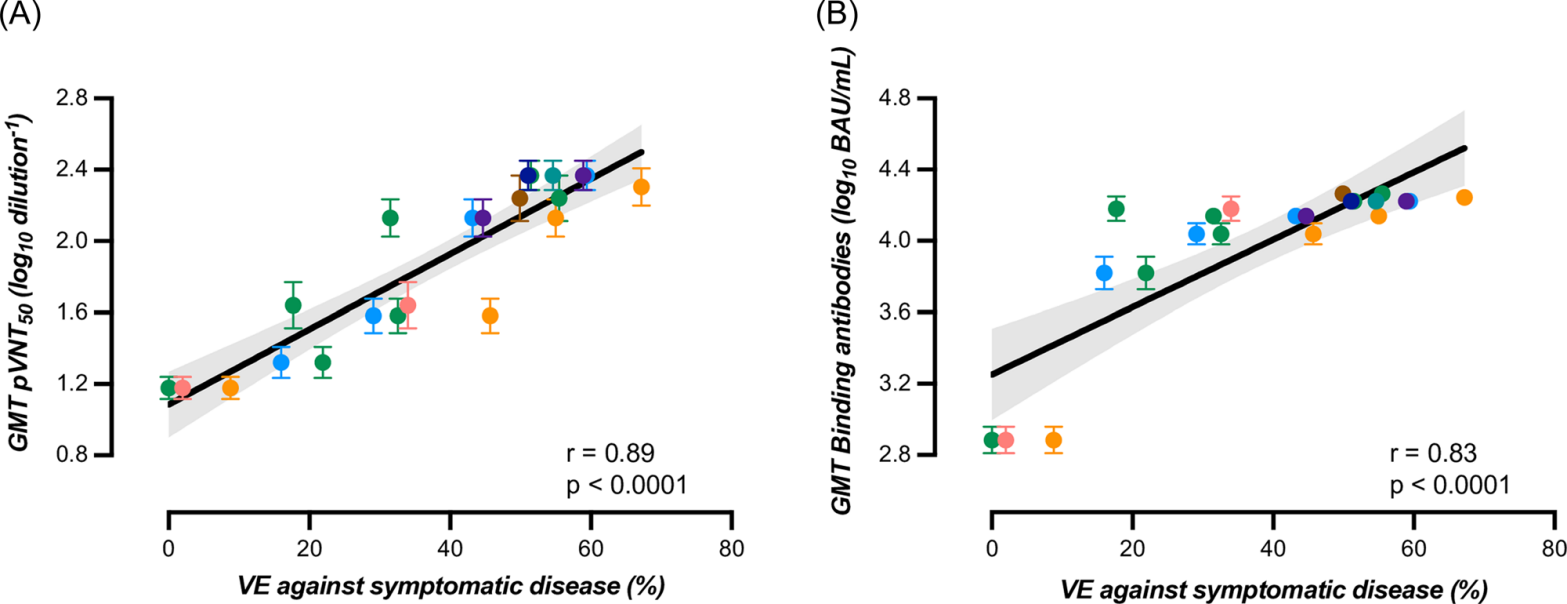
GMT (±95% CI) of (A) neutralizing antibodies and (B) binding antibodies against the vaccine efficacy against symptomatic disease (%). GMT from individuals with and without previous SARS‐CoV‐2 infection were merged. Vaccine efficacy (%) were gathered from the literature. Each color corresponds to a single study. CI, confidence interval; GMT, geometric mean titer

A significant correlation between binding antibodies and neutralizing titers was found (*r* = 0.51, 95% CI = 0.46–0.56, *p* < 0.0001) but the strength of agreement was null using the manufacturer's cut‐off of 0.8 BAU/ml since all results for binding antibodies were positive. Furthermore, there was a proportional and significant increase in binding antibodies according to categories of neutralizing titers. GMT for binding antibodies corresponding to neutralizing titer categories <20, 20–80, 81–160, 161–320, and >320 were 3286, 10 351, 12 481, 16 588, and 20 036 BAU/ml (Figure 4A). Based on the ROC curve analyses, an alternative cut‐off of 8434 BAU/ml for binding antibodies was identified to predict the neutralization of the Omicron BA.1 variant with a calculated sensitivity and specificity of 83.1% and 63.4%, respectively (area under the curve = 0.77, *p* < 0.0001) (Figure 4B). Therefore, there was a high proportion of sera (i.e., 36.6%) without neutralizing antibodies that had high titers of binding antibodies. Using this adapted cut‐off induced a Cohen's kappa of 0.45 (95% CI = 0.38–0.51) that corresponds to a moderate agreement. The GMT of binding and neutralizing antibodies obtained in our study correlated strongly with the VE (%) from symptomatic infection (*r* = 0.83 [95% CI = 0.63–0.93], *p* < 0.0001 and *r* = 0.89 [95% CI = 0.72–0.95], *p* < 0.001, for binding and neutralizing antibodies, respectively), with the respective equations: “y = 0.01890*x + 3.251” and “y = 0.02106*x + 1.085” (Figure 5).

## 4 DISCUSSION

Although homologous boosting with BNT162b2 vaccine elicited high titers of binding and neutralizing antibodies to Omicron BA.1 in the first weeks following vaccine administration, the response waned substantially within 6 months. The rapid increase in the humoral response was observed regardless of previous SARS‐CoV‐2 history. After 6 months, the decay of neutralizing antibodies was higher compared to binding antibodies (11.5‐ and 10.2‐fold decrease vs. 2.9‐ and 2.5‐fold‐decrease in COVID‐19 naive and in previously infected subjects, respectively). Accordingly, binding antibodies presented a significantly higher T_1/2_ (139–168 days) as compared to neutralizing antibodies (36–42 days). Nevertheless, the mean time to reach the neutralization cut‐off was similar, i.e., 186–194 days versus 182–214 days, for binding and neutralizing antibodies, respectively. The proportion of participants that was considered negative for binding and neutralizing antibodies was also related.

Interestingly, the global humoral response was not different in participants with a history of SARS‐CoV‐2 infection compared to COVID‐19 naive participants. This feature was not seen after the second dose for binding antibodies.^9^, ^49^ This is somewhat in contradiction with the study of Alatarwneh et al.^32^ who found that vaccination enhanced protection among persons who had had a previous SARS‐CoV‐2 infection. Hybrid immunity resulting from previous infection and recent booster vaccination conferred the strongest protection in this study.

Very few studies documented the long‐term kinetics of antibodies following the booster administration. After a follow‐up period of 3 months, Lyke et al.^20^ found a 3.5‐fold decrease in neutralizing antibodies against Omicron in a population of 50 participants having received the BNT162b2 booster. The observe decay was quite similar as in our study at 3 months (i.e., 5.5‐fold decrease). Munro et al.^50^ found a considerable decay of immunoglobulin G (IgG) titers 7 months after the homologous BNT162b2 booster in a small population of 31 COVID‐19 naive subjects (26 982 at 28 days and 3761 U/ml at 7 months: 7.2‐fold decrease). Neutralizing antibodies were not evaluated. Regev‐Yochay et al.^51^ also identified a decrease of IgG titers 4–5 months after the homologous booster administration (2102 at 28 days and 383 BAU/ml at 4–5 months resulting in a 5.5‐fold decrease). A parallel decay in neutralizing antibodies was also observed (2629 at 28 days and 480 BAU/ml at 4–5 months resulting in a 5.5‐fold decrease). Only COVID‐19 naive participants were included (*n* = 154). The waning of the humoral response observed in our study and in the literature for the postbooster period^50^, ^51^ was consistent with the one observed after the second dose of BNT162b2.^8^, ^9^ The distinct kinetics observed for IgG and total antibodies may be explained by the additional response of non‐IgG antibody isotypes, which may persist several months after vaccination.

The waning of antibodies over time after the BNT162 booster was proportional to the decrease of VE identified in the literature^7^, ^29^, ^30^, ^31^, ^33^, ^34^, ^42^, ^48^ and we found a stronger correlation between GMT of neutralizing antibodies (*r* = 0.89) compared to GMT of binding antibodies (*r* = 0.83), yet the difference was not significantly different. Previous studies focusing on primary vaccine schemes and mostly on the wild‐type virus identified that SARS‐CoV‐2 antibodies are a strong correlate of protection.^11^, ^12^, ^13^, ^14^ Lower levels of binding and neutralizing antibodies during the peri‐infection period were described in breakthrough patients in comparison to control patients (i.e., patients that did not develop infection), supporting the role of antibodies in protecting against infection.^52^, ^53^ Our results therefore reinforce the conclusions of these preliminary studies and show that these also applied to Omicron after the booster administration.

Although a waning of binding and neutralizing antibodies was observed as well as similar correlations between mean titers and VE, the two methods used to measure these antibodies were not commutable. Indeed, neutralizing antibodies, that represent a first layer of adaptive immunity against COVID‐19, were only modestly correlated (*r* = 0.51) against the commercial assay used. This latter was therefore not adapted to predict the presence of neutralizing antibodies. The refining of the cut‐off for binding antibodies at 8434 BAU/ml allowed us to improve the prediction of neutralizing antibodies, but the performance remained moderate since there is still a significant proportion of samples with high binding antibody titers that do not correspond to neutralizing activity against Omicron (Figure 3). Therefore, diagnostic companies should need to rethink their current commercial assays (i.e., modification of antigen and epitopes) to design assays capable of predicting neutralizing activity against emerging and highly mutated SARS‐CoV‐2 variants.^54^ This would also avoid any misinterpretation (i.e., high protection in case of high binding antibody titers).^54^ Methods used to measure neutralizing antibodies present a low throughput, are time‐consuming, need skillful operators, and require high levels of biosafety (especially for live virus neutralization assay).^54^ It would therefore be easier to use commercial assays that can be a surrogate of these reference methods.

In our study, we evaluated the vaccine‐induced neutralizing activity against Omicron BA.1. We were not able at that time to evaluate the neutralizing activity against Omicron sublineages BA.2, BA.2.12.1, BA.3, or BA.4/5. Lyke et al.^20^ identified similar neutralizing titers 29 days after the Moderna mRNA‐1273 booster between BA.1, BA.2, and BA.3, while a modest decline was observed for BA.2.12.1 (1.5‐fold) and BA.4/5 (2.5‐fold) in a total of 16 subjects. Accordingly, Hachmann et al.^26^ found no difference between BA.1 and BA.2, but significant lower titers for BA.2.12.1 (2.2‐fold) and BA.4/5 (3.3‐fold), 14 days after the BNT162b2 homologous booster in 27 participants. Cao et al.^25^ confirmed the same mean titers of neutralizing antibodies in 50 participants between BA.1 and BA.2 28 days after the CoronaVac homologous booster, and lower neutralizing activity compared to BA.1 for BA.2.12.1 (1.2‐fold) and BA.4/5 (1.6‐fold). However, Bowen et al.^55^ found similar neutralizing titers between BA.1, BA.2, BA.2.12.1 and B4/5, ±30 days after mRNA‐1273/BNT162b2 booster in 13 participants. A strong increase after the booster was also observed for Omicron sublineages.^20^, ^26^, ^55^ Our results 6 months after the booster might therefore be overestimated compared to BA.2.12.1 and B4/5 sublineages.

The administration of a fourth dose is currently under discussion^56^ and some interim recommendations have been formulated.^57^, ^58^ Considering the waning of VE over time and the considerable immune escape of new emerging variants, the fourth dose seems unavoidable to restore a sufficient level of neutralizing antibodies. The efficacy of a fourth dose (or second booster) against symptomatic disease, hospitalization and severe COVID‐19 has already been proved.^59^, ^60^, ^61^ The protection against confirmed infection, however, started to wane from 4 weeks since the fourth dose.^59^, ^61^ Magen et al.^59^ estimated a VE of 61% (95% CI = 58%–64%) against symptomatic disease 14–30 days after the fourth dose, which is consistent with the VE found 14–30 days after the third dose (57%; 95% CI = 51%–64%).^7^, ^30^, ^32^, ^33^, ^34^ The vaccine‐induced antibody titers after the fourth dose have been evaluated in few studies.^50^, ^51^ Two studies have enrolled 31 and 154 individuals and found a 7.8 and 11.4‐fold rise in IgG 14 days after the fourth BNT162b2 dose.^50^, ^51^ A consistent 10.7‐fold increase in neutralizing antibodies against Omicron was observed.^51^ This increase in neutralizing antibodies was similar to the one reported in our study 14 days after the third BNT162b2 dose (9.8‐ to 12.4‐fold increase). It was concluded that the maximal immunogenicity of mRNA vaccines that was achieved after three doses was similar compared to antibody levels generated after the fourth dose.^50^, ^51^ Taken all together, VE and antibody levels after the second booster were consistent with the ones after the first booster if considering the same time intervals since injection. Our linear model that could predict the level of VE according to neutralizing titers might therefore also be applicable for the fourth dose, but this deserves further validation.

The efficacy of the current formulation of the vaccine is at most around 65% against symptomatic disease 4 weeks after the administration of the first booster. On 25 June 2022, Pfizer and BioNTech announced that they are working on a Omicron‐adapted mRNA vaccine.^62^ On 31 August 2022, the U.S. Food and Drug Administration amended the emergency use authorization of the Moderna COVID‐19 Vaccine and the Pfizer‐BioNTech COVID‐19 Vaccine to authorize bivalent formulations of the vaccines. This bivalent vaccine, also called “updated booster,” contains two mRNA components of the virus (i.e., one original strain and one in common between the BA.4 and BA.5 lineages).^63^ This adapted version is expected to boost the protection against Omicron.

## 5 CONCLUSION

A rapid and significant increase in booster‐induced antibodies was observed from 7 days after the homologous BNT162b2 booster. Thereafter, a considerable antibody waning was noticed within 6 months, which was strongly correlated to VE data available in the literature. The impact of previous SARS‐CoV‐2 infection on the humoral response was nonsignificant after a first complete cycle of vaccination. Binding and neutralizing antibodies against Omicron followed a similar kinetics of decay but were only modestly correlated. A substantial increase of cut‐off for binding antibodies was needed to increase the prediction of a neutralizing activity. Nevertheless, there was still a considerable number of participants with high binding antibody titers that did not present any neutralizing capacity. Commercial assays available in clinical laboratories might therefore require adaptation to better predict neutralizing antibodies, which represent the best correlate of protection. Our kinetic models might also be useful to determine the timing of fourth dose administration.

## AUTHOR CONTRIBUTIONS

*Conceptualization*: Julien Favresse and Jonathan Douxfils. *Methodology*: Julien Favresse, Constant Gillot, Jean‐Louis Bayart, Clara David, Germain Simon, Loris Wauthier and Mélanie Closset. *Software*: Julien Favresse, Constant Gillot, and Jonathan Douxfils; *Validation*: Julien Favresse and Jonathan Douxfils. *Formal analysis*: Julien Favresse, Constant Gillot, and Jonathan Douxfils. Investigation: Julien Favresse, Constant Gillot, Jean‐Louis Bayart and Jonathan Douxfils. *Resources*: Jean‐Michel Dogné and Jonathan Douxfils. *Data curation*: Julien Favresse, Constant Gillot, Jean‐Louis Bayart, Clara David, Germain Simon, Loris Wauthier, and Mélanie Closset. *Writing—original draft preparation*: Julien Favresse. *Writing—review and editing*: Julien Favresse, Constant Gillot, Jean‐Louis Bayart, Loris Wauthier, Jean‐Michel Dogné, Jonathan Douxfils. *Supervision*: Jonathan Douxfils. *Project administration*: Julien Favresse, Jean‐Louis Bayart, Mélanie Closset, and Jonathan Douxfils. *Funding acquisition*: Julien Favresse, Jean‐Louis Bayart, Mélanie Closset, Jean‐Michel Dogné, and Jonathan Douxfils. All authors have read and agreed to the published version of the manuscript.

## CONFLICTS OF INTEREST

The authors declare no conflicts of interest.

## Supporting information

Supplementary information.

## Data Availability

The data presented in this study are available on request from the corresponding author. The data are not publicly available according to the ethical committee decision on the conduct of this study.
