# Supplementary material for: Vaccine‐induced binding and neutralizing antibodies against Omicron 6 months after a homologous BNT162b2 booster
Source: J Med Virol. 2022 Oct 1:10.1002/jmv.28164. Online ahead of print. doi: 10.1002/jmv.28164 (PMC9538323; doi:10.1002/jmv.28164)
Supplement: Supplementary file 1 — Supplementary information. [file JMV-9999-0-s001.docx]

|  | Namur, Belgium  September 02, 2022  *Journal of Medical Virology* | |
| --- | --- | --- |
|  | |  |

Dear Dr. Shou-Jiang Gao

We are pleased to submit for consideration to the *Journal of Medical Virology*, our revised paper entitled:

**“Vaccine-induced binding and neutralizing antibodies against Omicron 6 months after a homologous BNT162b2 booster (JMV-22-16772)”.**

Our responses to reviewers’ comments are presented below. Statements about data availability, conflict of interest statement and author contribution have been added in the main document. We also added a new reference at the end of the discussion dealing about the future release of the “updated booster” that contains two mRNA components of the virus (i.e., one original strain and one in common between the BA.4 and BA.5 lineages) (*https://www.fda.gov/news-events/press-announcements/coronavirus-covid-19-update-fda-authorizes-moderna-pfizer-biontech-bivalent-covid-19-vaccines-use*). We also took the liberty to modify the layout of the figures 3 and 4B in order to be more in line with the others.

This data has not been previously published and is not under consideration for publication elsewhere.

All authors approved the final version of the manuscript.

We look forward to hearing from you soon.

Yours sincerely,

Julien Favresse,

On behalf of the authors of this study.

**Reviewing: 1**

Comments to the Author (blind)
The novelty of the research topic is too limited.

We would like to thank the reviewer for the time taken to read our article. We are sorry to hear that the reviewer found that the novelty of our research was too limited. If fact, we think that our results are among the first to document the humoral kinetics of both the binding and neutralizing antibodies against Omicron over a period of 6 months. The kinetic models used, the correlation between binding antibodies and NAbs as well as the fact that we proved that the levels of antibodies is still correlated to vaccine efficacy during Omicron era represent some degree of novelty for us, in regards to the current literature.

**Reviewing: 2**

Comments to the Author (blind)
The present study provides critical evidence on the antibody duration, in terms of binding affinity and neutralization activity against Omicron BA.1 variant, induced by BNT162b2 booster. Although the protection by BNT162b2 has already been extensively studied, the long-term persistence of humoral immune is still need to be illustrated, and this is imperative because SARS-CoV-2 will coexist for a long time. The authors of this study determined binding antibodies and neutralizing antibodies titers of 155 healthcare workers and analyzed the kinetics of these antibodies, finding that neutralizing antibodies, as well as binding antibodies, waned significantly over 3 months and this decrease is not affected by the previous infection of SARS-CoV-2. These results are consistent with recent studies mentioned in the manuscript. In addition, the fourth dose and the Omicron-adapted new vaccine are discussed based on the quickly waning of BNT162b2 third booster induced antibody observed in the present study. Generally, this is a very informative and significant study and analysis made by the authors are very solid, and the conclusion is helpful for the future strategy against COVID-19 pandemic.

We thank the reviewer for this positive comment.

Here are few minor questions
The article would be more readable if the each T1/2 and Tmax of neutralizing and binding antibodies in SARS-CoV-2 naive or infected individual are marked in figures.

We thank the reviewer for this suggestion. The figure 3 has been adapted. We agree that the paper will be more readable with this modification.

Line 208. To keep the expression consistent in the context, “11.0 days” should be “11 days”.

The sentence has been adapted accordingly. T_1/2_ and T_max_ were added on the figures.

Line 187. Whether the half-life of antibodies in previous infected individual is “binding antibodies” or “neutralizing antibodies”? 
Sorry for this mistake. It concerns neutralizing antibodies. The sentence has been adapted accordingly. Thank you.

Reference update
Recently, Sneh et.al published an article in JV evaluated the BNT162b2 induced humoral response in terms of people at different ages. This article should be cited in the present study. 
Gupta SL, Mantus G, Manning KE, Ellis M, Patel M, Ciric CR, Lu A, Turner JS, O'Halloran JA, Presti RM, Joshi DJ, Ellebedy AH, Anderson EJ, Rostad CA, Suthar MS, Wrammert J. Loss of Pfizer (BNT162b2) Vaccine-Induced Antibody Responses against the SARS-CoV-2 Omicron Variant in Adolescents and Adults. J Virol. 2022 Aug 17:e0058222. doi: 10.1128/jvi.00582-22. Epub ahead of print. PMID: 35976000.

The reference has been included in the article. Thank you for the suggestion.

**EDITORIAL OFFICE COMMENTS**
When submitting your revision, please note that we will require some extra information from you. If this information is not supplied at revision, it may result in the delay in processing of your manuscript:
•       Data Availability Statement
•       Conflict of Interest statement
•       Author contribution statement
